# Supplementary material for: Comprehensive transcriptome analysis provides new insights into nutritional strategies and phylogenetic relationships of chrysophytes
Source: PeerJ. 2017 Jan 10;5:e2832. doi: 10.7717/peerj.2832 (PMC5228505; doi:10.7717/peerj.2832)
Supplement: Table S1 — Significantly differentially expressed genes between heterotrophic and mixotrophic organisms. Shown are the KEGG Orthology ID (KO), gene symbol and name, log2 fold-change, p-value and Benjamini and Hochberg (BH) adjusted p-value. [file peerj-05-2832-s001.pdf]

KO	symbol	gene name	log2FC	p-value	adj. p-value
K08906	petJ	cytochrome c6	-12.37	2.96E-14	3.82E-11
K02636	petC	cytochrome b6-f complex iron-sulfur subunit [EC:1.10.9.1]	-13.56	1.96E-13	1.26E-10
K00218	E1.3.1.33, por	protochlorophyllide reductase [EC:1.3.1.33]	-11.21	1.68E-07	5.43E-05
K03428	E2.1.1.11, chlM, bchM	magnesium-protoporphyrin O-methyltransferase [EC:2.1.1.11]	-11.24	1.65E-07	5.43E-05
K00463	INDO	indoleamine 2,3-dioxygenase [EC:1.13.11.52]	8.56	3.46E-07	8.92E-05
K01768	E4.6.1.1	adenylate cyclase [EC:4.6.1.1]	-10.36	1.47E-06	3.16E-04
K09839	VDE, NPQ1	violaxanthin de-epoxidase [EC:1.10.99.3]	-8.86	3.31E-06	6.10E-04
K15634	gpmB	probable phosphoglycerate mutase [EC:5.4.2.12]	8.22	4.10E-06	6.61E-04
K11150	RDH8	retinol dehydrogenase 8 [EC:1.1.1.-]	-10.29	4.82E-06	6.91E-04
K03002	RPA2, POLR1B	DNA-directed RNA polymerase I subunit RPA2 [EC:2.7.7.6]	8.24	5.76E-06	7.44E-04
K14424	SMO2	4-alpha-methyl-delta7-sterol-4alpha-methyl oxidase [EC:1.14.13.72]	8.17	1.05E-05	1.18E-03
K09838	ZEP, ABA1	zeaxanthin epoxidase [EC:1.14.13.90]	-8.66	1.09E-05	1.18E-03
K00284	E1.4.7.1	glutamate synthase (ferredoxin) [EC:1.4.7.1]	-8.82	1.19E-05	1.18E-03
K10259	MET30	F-box and WD-40 domain protein MET30	-9.28	3.64E-05	3.36E-03
K02690	psaB	photosystem I P700 chlorophyll a apoprotein A2	-7.45	4.72E-05	3.83E-03
K02257	COX10	protoheme IX farnesyltransferase [EC:2.5.1.-]	8.18	4.79E-05	3.83E-03
K01275	CTSC	cathepsin C [EC:3.4.14.1]	-9.13	5.05E-05	3.83E-03
K07253	MIF	phenylpyruvate tautomerase [EC:5.3.2.1]	-9.03	6.91E-05	4.96E-03
K09565	PPIF	peptidyl-prolyl isomerase F (cyclophilin D) [EC:5.2.1.8]	7.60	1.48E-04	8.29E-03
K00616	E2.2.1.2, talA, talB	transaldolase [EC:2.2.1.2]	6.83	1.42E-04	8.29E-03
K03527	ispH, lytB	4-hydroxy-3-methylbut-2-enyl diphosphate reductase [EC:1.17.1.2]	-7.54	1.32E-04	8.29E-03
K02720	psbV	photosystem II cytochrome c550	-8.62	1.38E-04	8.29E-03
K17705	KRIT1	Krev interaction trapped protein 1	-8.63	1.34E-04	8.29E-03
K00223	ERG4	delta24(24(1))-sterol reductase [EC:1.3.1.71]	7.41	1.62E-04	8.71E-03
K16054	DEP1	methylthioribulose 1-phosphate dehydratase / enolase-phosphatase E1 [EC:4.2.1.109 3.1.3.77]	7.04	1.84E-04	8.84E-03
K03938	NDUFS5	NADH dehydrogenase (ubiquinone) Fe-S protein 5	6.87	1.78E-04	8.84E-03
K15744	Z-ISO	zeta-carotene isomerase [EC:5.2.1.12]	-8.54	1.85E-04	8.84E-03
K00927	PGK, pgk	phosphoglycerate kinase [EC:2.7.2.3]	-5.16	2.90E-04	1.34E-02
K11927	rhlE	ATP-dependent RNA helicase RhlE [EC:3.6.4.13]	7.04	3.12E-04	1.39E-02
K00134	GAPDH, gapA	glyceraldehyde 3-phosphate dehydrogenase [EC:1.2.1.12]	-5.21	3.83E-04	1.65E-02

K02293	PDS, crtP	15-cis-phytoene desaturase [EC:1.3.5.5]	-6.81	3.99E-04	1.66E-02
K02707	psbE	photosystem II cytochrome b559 subunit alpha	-6.87	5.50E-04	2.22E-02
K08681	pdxT, pdx2	5'-phosphate synthase pdxT subunit [EC:4.3.3.6]	-6.33	7.42E-04	2.90E-02
K00031	IDH1, IDH2, icd	isocitrate dehydrogenase [EC:1.1.1.42]	5.13	7.67E-04	2.91E-02
K00416	QCR6, UQCRH	ubiquinol-cytochrome c reductase subunit 6	-6.52	7.93E-04	2.92E-02
K10257	FAD8, desB	omega-3 fatty acid desaturase (delta-15 desaturase) [EC:1.14.19.-]	7.06	9.11E-04	3.27E-02
K11827	AP2S1	AP-2 complex subunit sigma-1	6.40	9.88E-04	3.45E-02
K02132	ATPeF1A, ATP5A1, ATP1	F-type H+-transporting ATPase subunit alpha	3.74	1.30E-03	4.21E-02
K03100	lepB	signal peptidase I [EC:3.4.21.89]	6.47	1.34E-03	4.21E-02
K08239	GNPTAB	UDP-N-acetylglucosamine-lysosomal-enzyme [EC:2.7.8.17]	6.29	1.25E-03	4.21E-02
K08901	psbQ	photosystem II oxygen-evolving enhancer protein 3	-6.50	1.30E-03	4.21E-02
K08334	BECN1, VPS30, ATG6	beclin 1	6.52	1.56E-03	4.80E-02
K10144	RCHY1, PIRH2	RING finger and CHY zinc finger domain-containing protein 1 [EC:6.3.2.19]	6.37	1.64E-03	4.94E-02
K03404	chlD, bchD	magnesium chelatase subunit D [EC:6.6.1.1]	-7.03	1.88E-03	5.51E-02
K13953	adhP	alcohol dehydrogenase, propanol-preferring [EC:1.1.1.1]	-6.03	1.93E-03	5.54E-02
K06134	COQ7	ubiquinone biosynthesis monooxygenase Coq7 [EC:1.14.13.-]	6.30	1.97E-03	5.54E-02
K03130	TAF5	transcription initiation factor TFIID subunit 5	6.39	2.48E-03	6.16E-02
K07366	GRAP2, GADS	GRB2-related adaptor protein 2	6.48	2.45E-03	6.16E-02
K05641	ABCA1	ATP-binding cassette, subfamily A (ABC1), member 1	-5.38	2.40E-03	6.16E-02
K09518	DNAJB12	DnaJ homolog subfamily B member 12	5.43	2.48E-03	6.16E-02
K11129	NHP2, NOLA2	H/ACA ribonucleoprotein complex subunit 2	5.32	2.26E-03	6.16E-02
K11094	SNRPB2	U2 small nuclear ribonucleoprotein B"	-6.91	2.31E-03	6.16E-02
K14157	AASS	alpha-aminoadipic semialdehyde synthase [EC:1.5.1.8 1.5.1.9]	6.32	2.56E-03	6.25E-02
K03952	NDUFA8	NADH dehydrogenase (ubiquinone) 1 alpha subcomplex subunit 8	6.00	2.81E-03	6.43E-02
K02113	ATPF1D, atpH	F-type H+-transporting ATPase subunit delta	6.27	2.83E-03	6.43E-02
K13998	DHFR-TS	dihydrofolate reductase / thymidylate synthase [EC:1.5.1.3 2.1.1.45]	5.98	2.84E-03	6.43E-02
K12948	SPCS3, SPC3	signal peptidase complex subunit 3 [EC:3.4.-.-]	5.98	2.78E-03	6.43E-02
K00365	E1.7.3.3	urate oxidase [EC:1.7.3.3]	6.26	3.56E-03	7.79E-02
K02974	RP-S24e, RPS24	small subunit ribosomal protein S24e	4.70	3.53E-03	7.79E-02

K01581	E4.1.1.17, ODC1, speC, speF	ornithine decarboxylase [EC:4.1.1.17]	4.59	3.87E-03	8.18E-02
K02716	psbO	photosystem II oxygen-evolving enhancer protein 1	-5.98	3.81E-03	8.18E-02
K01809	manA, MPI	mannose-6-phosphate isomerase [EC:5.3.1.8]	-5.67	4.21E-03	8.76E-02
K10413	DYNC1H	dynein heavy chain 1, cytosolic	-4.91	4.74E-03	9.71E-02
K04077	groEL, HSPD1	chaperonin GroEL	-3.80	4.87E-03	9.83E-02
K13025	EIF4A3, FAL1	ATP-dependent RNA helicase [EC:3.6.4.13]	-3.39	5.16E-03	9.94E-02
K02897	RP-L25, rplY	large subunit ribosomal protein L25	5.92	5.10E-03	9.94E-02
K06066	CIR	CBF1 interacting corepressor	5.52	5.15E-03	9.94E-02
