# Supplementary figures and images for: Comprehensive transcriptome analysis provides new insights into nutritional strategies and phylogenetic relationships of chrysophytes
Source: PeerJ. 2017 Jan 10;5:e2832. doi: 10.7717/peerj.2832 (PMC5228505; doi:10.7717/peerj.2832)

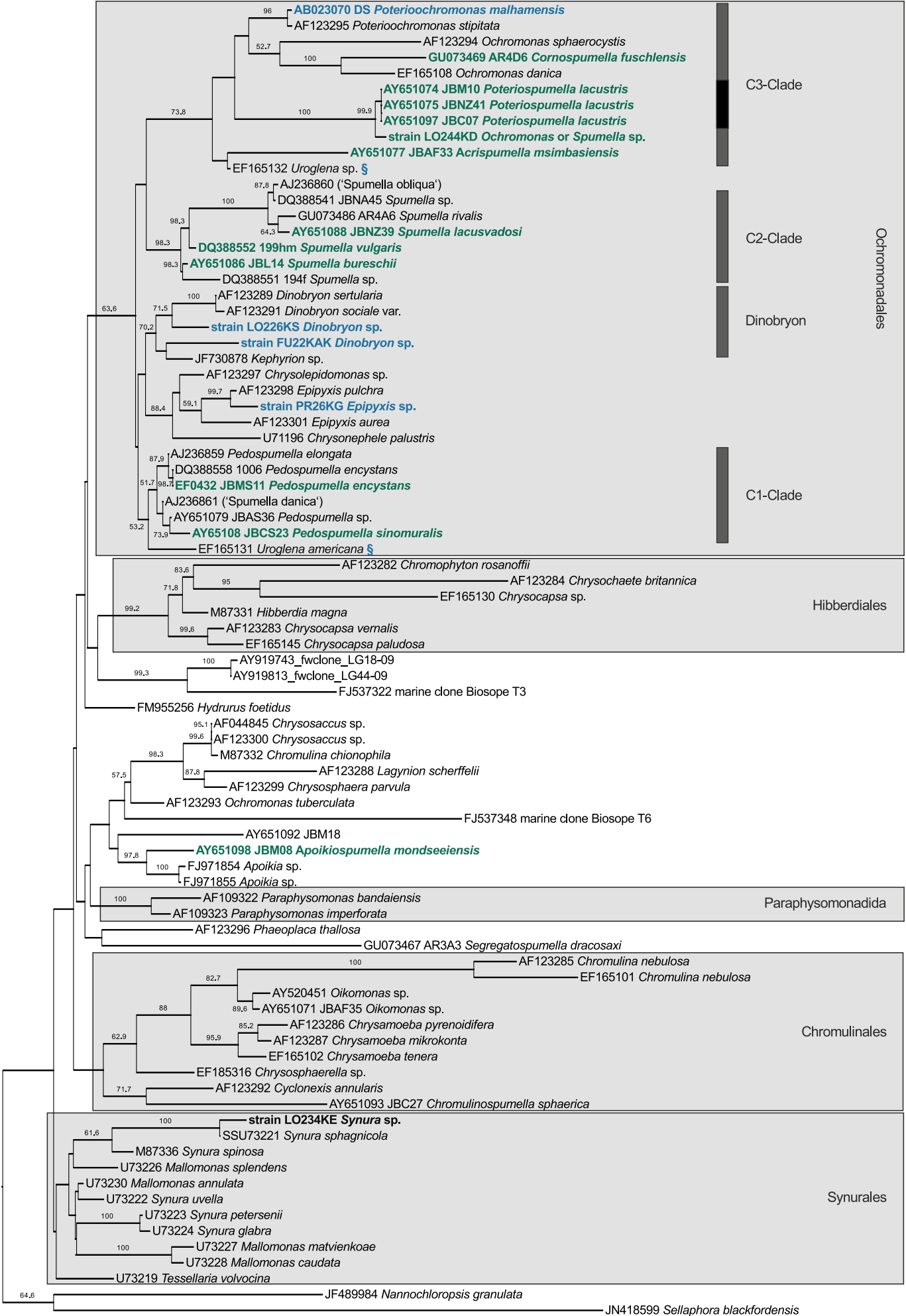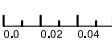

Supplement: Figure S1 — Maximum-likelihood phylogeny based on SSU sequences showing the investigated strains (bold print, coloured according to trophic mode as in Figure 1) within Chrysophyceae. Numbers at nodes are bootstrap values (values exceeding 50% are shown). The paragraph sign (§) depicts two possible possitions of the Uroglena species strain WA34KE, whose SSU was not sequenced yet. [file peerj-05-2832-s002.pdf]
